# Direct and indirect comparisons in network meta-analysis of SuperPATH, direct anterior and posterior approaches in total hip arthroplasty

**DOI:** 10.1038/s41598-022-20242-3

**Published:** 2022-10-06

**Authors:** Nikolai Ramadanov, Simon Bueschges, Kuiliang Liu, Philip Lazaru, Ivan Marintschev

**Affiliations:** 1 Center of Orthopaedics and Traumatology, University Hospital Brandenburg an der Havel, Brandenburg Medical School Theodor Fontane, Neuruppin, Germany; 2 Faculty of Medicine, Department of Statistics, University of Salamanca, Salamanca, Spain; 3 Department for Orthopaedics and Trauma Surgery, Siloah St. Trudpert Hospital, Pforzheim, Germany; 4 Center for Surgery, Evangelical Hospital Ludwigsfelde-Teltow, Ludwigsfelde, Germany; 5 Department of Trauma, Hand and Reconstructive Surgery, University Hospital Jena, Friedrich Schiller University, Jena, Germany

**Keywords:** Randomized controlled trials, Fracture repair, Medical research

## Abstract

SuperPATH is a novel approach to the hip joint that needs to be compared to other known surgical approaches. To conduct a network meta-analysis (NMA) of randomized controlled trials (RCTs) comparing short-term outcomes of SuperPATH, direct anterior (DAA), and posterior/ posterolateral approaches (PA) in total hip joint arthroplasty (THA). We performed a systematic review on PubMed, CNKI, Embase, The Cochrane Library, Clinical trials, and Google Scholar up to November 30th, 2021. We assessed treatment effects between SuperPATH, DAA, and PA by performing a frequentist NMA, including a total of 20 RCTs involving 1501 patients. SuperPATH showed a longer operation time (MD = 16.99, 95% CI 4.92 to 29.07), a shorter incision length (MD = −4.71, 95% CI −6.21 to −3.22), a lower intraoperative blood loss (MD = −81.75, 95% CI  −114.78 to −48.72), a higher HHS 3, 6 and 12 months postoperatively (MD = 2.59, 95% CI 0.59–4.6; MD = 2.14, 95% CI 0.5–3.77; MD = 0.6, 95% CI 0.03–1.17, respectively) than PA. DAA showed a higher intraoperative blood loss than PA and SuperPATH (MD = 91.87, 95% CI  27.99–155.74; MD = 173.62, 95% CI 101.71–245.53, respectively). No other relevant differences were found. In conclusion, the overall findings suggested that the short-term outcomes of THA through SuperPATH were statistically superior to PA. DAA and PA as well as SuperPATH and DAA showed indifferent results.

## Introduction

Patient outcomes after hip arthroplasty have improved over the past few decades. SuperPATH was introduced by Chow in 2011 as a novel hip approach in an attempt to solve the remaining problems^1^. Benefits of SuperPATH: Hip surgery in situ with the lower limb resting throughout the whole operation; tissue-sparing dissection through the interval between the gluteus medius and piriformis muscles; joint capsule preservation; unproblematic exposure of the acetabulum through accessory access. Two network meta-analyses (NMAs) comparing SuperPATH, the direct anterior approach (DAA), and conventional approaches showed some advantages of SuperPATH^2,3^. The strong limitation of those two NMAs was that they summarized all conventional approaches, although these differ significantly in terms of surgical techniques and outcome^4–6^. A different methodological approach is required to overcome this limitation. We performed another NMA of randomized controlled trials (RCTs) with the following PICO (Population, Intervention, Control, and Outcomes) question: In patients with hip disease or fracture, is the short-term outcome after total hip arthroplasty (THA) implanted through SuperPATH better compared to DAA and/or posterior/posterolateral (posterior/posterolateral approach = PA)?

## Methods

### Details on the SuperPATH technique

The SuperPATH technique is briefly described as follows: the incision of the capsule is performed through a 6–10 cm skin incision and a muscle-sparing approach between the piriformis and gluteus minimus muscles in lateral decubitus position. The femoral canal is then opened with a reamer, the femur is broached and osteotomy of the femoral neck is performed. Following exposure of the acetabulum, the acetabular reamers are inserted through the main incision and attached within the surgical field to the shaft of the motorized drill, which is inserted through a separate percutaneous portal passing adjacent to the posterior aspect of the proximal femur. After implantation of the cup, inlay, modular neck, and head, reposition is performed. Wound closure concludes the operation.

### Search strategy and study selection

The PRISMA recommendations were followed when performing and presenting our NMA^7^. The review protocol was registered in PROSPERO on September 11th, 2021 (CRD42021272994). Our search strategy and methods were similar to our previous works^2,3^. We searched the following databases and checked citations of screened studies and related meta-analyses for relevant manuscripts up to November 30th, 2021, without restrictions to publication date or language: PubMed, China National Knowledge Infrastructure (CNKI), Embase, The Cochrane Library, Clinical trials. We built a BOOLEAN search strategy for RCTs on SuperPATH and DAA as follows: [(SuperPATH OR supercapsular percutaneously assisted approach in total hip arthroplasty)] and [(THR OR THA OR total hip arthroplasty) AND (approach) AND (anterior OR posterior OR posterolateral)]. We adapted search terms to the syntax of the used databases. Furthermore, we searched Google Scholar for relevant RCTs. Titles, abstracts, and then full-text articles were independently reviewed by two reviewers (NR and PL). The decision on the inclusion of each study was determined by the consensus between the two reviewers. Cases of disagreement were resolved by discussion with a third reviewer (KL). Kappa coefficient was used to measure the agreement between the reviewers. The entire search and selection process was carried out separately for studies on DAA and studies on SuperPATH, using the same methods. A Chinese-speaking reviewer (KL) helped with the search in CNKI.

### Inclusion/exclusion criteria

We included RCTs with human participants with hip disease or hip fracture, who had THA through either DAA or SuperPATH compared to PA. We excluded studies for the following reasons: no outcome of interest, employment of a computer navigation system, and hip replacement with hemiarthroplasty.

### Types of outcome measures

1. Surgical outcome
  The operation time (in min.) was defined as the time interval from the skin incision to suture. It correlates with the operating skills and with the risk of infection.
  The incision length (in cm) was measured on a graduated scale. It is one of several indicators of intraoperative trauma.
  The intraoperative blood loss (in ml) was defined as the total amount of blood from the suction device. It is an indirect indicator of intraoperative trauma.
2. Functional outcome
  The Harris Hip Score (HHS) was developed for the assessment of the results of hip surgery^8^. The hip joint function was periodically evaluated at 3, 6, and 12 months postoperatively. The score adds points from the evaluation of four categories: pain, function, degree of deformity, and hip range of motion. The best achievable score is 100 points.
3. Radiological outcome
  The acetabular cup anteversion angle and the inclination angle (in degrees) have ideal values for positioning: anteversion angle from 10° to 25° and inclination angle from 40° to 50°^9^. A too large anteversion angle often leads to posterior impingement, resulting in anterior dislocation, and a too small anteversion angle leads to posterior dislocation.

### Data extraction and quality assessment

Two reviewers (NR and PL) extracted the following relevant data into a data extraction form in a standard electronic spreadsheet and the Cochrane software program Review Manager Version 5.3: first author, year of publication, number of patients, patient characteristics, risk of bias and outcome. Cases of disagreement were resolved by discussion with a third reviewer (KL). The assessment of the risk of bias and the level of evidence was carried out independently by two reviewers (NR and KL) according to Cochrane's Risk of Bias 2 (RoB 2) tool^10^, respectively according to the recommendations of the GRADE system^11^.

### Statistical analysis

#### Direct comparison: measures of treatment effect

SuperPATH or DAA represented the “experimental group” and PA represented the “control group”. A direct comparison with both fixed and random effects models was applied to calculate the results for either SuperPATH or DAA and PA. We limited the presentation of statistical calculations to random effects model, as this method was more conservative and provided better estimates with wider confidence intervals. Mean differences (MDs) with 95% confidence intervals (CIs) were estimated for all outcomes. A common τ^2^ was assumed for calculation of the estimates of the random effects, using the DerSimonian and Laird method. Study weighting was performed by inverse variance^12^. In our NMA, we adhered to the Cochrane Handbook for Systematic Reviews of Interventions^13^.

#### Indirect comparison: network meta-analysis

A NMA using frequentist methods^14^ was performed, borrowing information from the direct comparisons mentioned above and using the PA group as a common comparator and reference node within the network. The following programs were used: meta and netmeta^15^. In addition, we calculated prediction intervals to estimate where to expect the results of future NMAs. We presented both direct comparison estimates and network estimates in a forest plot per outcome on a common scale. All statistic calculations were performed by a professional statistician (SB).

### Assessment of heterogeneity

We assessed heterogeneity with a test on Cochrane’s Q statistic, which followed a distribution with k-degrees of freedom (p-value < 0.10 is indicative of heterogeneity), and with a Higgins’ test I^2^ (low heterogeneity, < 25%; moderate heterogeneity, 25–75%; and high heterogeneity, > 75%)^16^. Results were presented regardless of the detection of heterogeneity to maintain the informative value within the forest plots. We did not pool study data that were clinically too diverse.

## Results

### Study identification and selection

**Figure 1:**
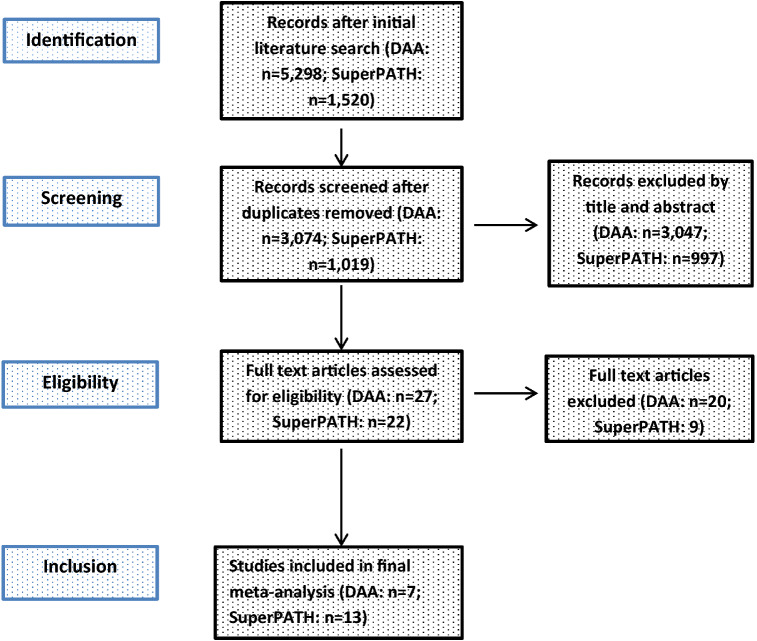
PRISMA flow diagram of the search results and selection according to our inclusion criteria.

A description of the study selection process is presented in a PRISMA flow diagram (Fig. 1). A total of 1019 studies were identified in our initial literature search on SuperPATH, after removing 501 duplicates. After the first screening procedure by title and abstract (κ = 1.0) with total agreement by the reviewers, 22 RCTs were assessed for eligibility. After the second screening procedure by full-paper analysis (κ = 1.0), 13 RCTs on SuperPATH^17–19,19–28^ were included in the final NMA. A total of 3074 studies were identified in our initial literature search on DAA, after removing 2251 duplicates. After the first screening procedure by title and abstract (κ = 0.96) with disagreement between the reviewers concerning 1 RCT, 27 RCTs were assessed for eligibility. After the second screening procedure by full-paper analysis (κ = 1.0), 7 RCTs on DAA^29–35^ were included in the final NMA.

### Characteristics of the RCTs

Thirteen RCTs, published between 2017 and 2021, compared SuperPATH with PA, altogether including 919 patients (with 923 operated hip joints). Of those patients, 459 were operated through SuperPATH and 460 through PA. The sample size of those RCTs ranged from 4 to 116 patients. Four RCTs were published in English language^19–21,25^, five RCTs in Chinese with an English abstract^22,23,26–28^ and four RCTs only in Chinese^17–19,24^. Seven RCTs, published between 2013 and 2020, compared DAA with PA, altogether including 582 patients. Of those patients, 291 were operated through DAA and 291 through PA. The sample size of those RCTs ranged from 46 to 120 patients. All RCTs on DAA were published in English language. Of the 7 RCTs included, 4 RCTs reported having used a traction table in THA through DAA^29–32^. The main characteristics of the 20 RCTs on SuperPATH and DAA with an overall 1501 included patients are presented in Table 1.

**Table 1:** Main characteristics of RCTs included in network meta-analysis.

	Sample size, n		Surgical approach		Mean age, y (SD or range)		Gender (M/F), n		BMI, kg/m^2^ (SD or range)		Imaging procedure for acetabular cup positioning	Hip pathology			
SuperPATH															
Study	Pts	Hips	S	PA	S	PA	S	PA	S	PA	S/PA	Osteoarthritis	ANFH	Dysplasia	Fracture
Gao and Shi^17^	70	70	35	35 P	69.26 ± 3.28	68.81 ± 3.45	23/12	20/15	23.09 ± 2.57	23.21 ± 2.44	Not reported	–	–	–	70
Li^18^	60	60	30	30 PL	70.35 ± 4.26	70.12 ± 4.78	16/14	18/12	Not reported	Not reported	Not reported	Not reported			
Li et al.^19^	96	96	49	47 PL	75.53 ± 7.34	77.21 ± 7.84	27/22	24/23	22.99 ± 2.87	22.7 ± 3	Not reported	Not reported			
Liu et al.^20^	94	94	47	47 PL	68.27 ± 3.71	68.55 ± 3.4	26/21	24/23	Not reported	Not reported	Not reported	–	–	–	94
Meng et al.^21^	4	8	4	4 PL	51.00 ± 4.54		4/0		21.49 (19.60–23.04)		Conv. X-Rays	–	8	–	–
Meng et al.^22^	40	40	20	20 Mini-PL	64.55 ± 9.06	65.25 ± 10.33	8/12	9/11	23.36 ± 2.55	22.82 ± 2.61	Conv. X-Rays	40	–	–	–
Ouyang et al.^23^	24	24	12	12 PL	54 (45–71)	55 (47–67)	8/4	9/3	23.1 (17.5–26.7)	23.9 (16.9–30.4)	Conv. X-Rays	11	13	–	–
Pan et al.^24^	116	116	58	58 PL	65.23 ± 6.84	65.62 ± 6.96	34/24	33/25	22.24 ± 4.15	22.56 ± 4.22	Not reported	23	33	9	51
Wang and Ge^25^	85	85	43	42 PL	71.53 ± 3.76	71.58 ± 3.79	26/17	24/18	22.47 ± 1.12	22.51 ± 1.15	Not reported	–	–	–	85
Xie et al.^26^	92	92	46	46 P	66.6 ± 11.88	64.47 ± 12.09	12/34	19/27	23.62 ± 1.63	24.06 ± 2.72	Conv. X-Rays	–	–	–	92
Yuan et al.^27^	84	84	40	44 PL	74.3 (67–79)	75.7 (69–82)	24/16	21/23	22.73 ± 1.71	22.36 ± 1.89	Not reported	11	22	6	45
Zhang et al.^28^	54	54	27	27 PL	62.41 ± 6.44	61.28 ± 6.7	10/17	12/15	24.53 ± 5.31	23.93 ± 4.89	Not reported	16	29	9	–
Zunlong et al.^29^	100	100	50	50 PL	89.14 ± 3.6	88.95 ± 3.71	31/19	29/21	Not reported	Not reported	Not reported	–	–	–	–

DAA															
Study	Pts	Hips	DAA	PA	DAA	PA	DAA	PA	DAA	PA	DAA/PA	Osteoarthritis	ANFH	Dysplasia	Fracture
Barrett et al.^30^	87	87	43TT	44 PL	61.4 ± 9.2	63.2 ± 7.7	29/14	19/25	30.7 ± 5.4	29.1 ± 5	Conv. X-Rays	87	–	–	–
Bon et al.^31^	100	100	50 TT	50 PL	67.26 ± 10	68.98 ± 7.93	21/29	23/27	26.46 ± 3.58	26.69 ± 3.12	Conv. X-Rays	100	–	–	–
Cheng et al.^32^	73	73	35TT	38 P	59	62.5	15/20	18/20	27.7	28.3	Conv. X-Rays	73	–	–	–
Moerenhout et al.^33^	55	55	28 TT	27 P	70.4 ± 9.1	68.9 ± 8.8	11/17	18/9	27.6 ± 4.4	26.5 ± 4.3	Conv. X-Rays	55		–	–
Rykov et al.^34^	46	46	23	23 PL	62.8 ± 6.1	60.2 ± 8.1	8/15	11/12	29 ± 5.6	29.3 ± 4.8	Not reported	46	–	–	–
Taunton et al.^35^	101	101	52	49 Mini-P	65 ± 10	64 ± 11	27/25	25/24	29 ± 22	30 ± 4	Conv. X-Rays	101	–	–	–
Zhao et al.^36^	120	120	60	60 PL	64.88 ± 12.13	62.18 ± 14.72	24/36	26/34	24.35 ± 3.1	25.58 ± 2.83	Conv. X-Rays	81	26	13	–

DAA: direct anterior approach; S: SuperPATH; TT: traction table PL: posterolateral approach; P: posterior approach; Pts: patients; Conv. = conventional.

### Risk of bias and level of evidence

The quality of the included RCTs was assessed according to the Cochrane Collaboration’s tool for risk of bias (Table 2) and according to the recommendations of the GRADE system (Table 3).

**Table 2:** Risk of bias assessment.

Study	Bias arising from the randomization process	Bias due to deviation from intended interventions		Bias due to missing outcome data	Bias in measurement of the outcome	Bias in selection of the reported result	Overall risk of bias
**SuperPATH vs. PA**							
Gao and Shi^17^	+	?		−	+	+	−
Li^18^	+	?		−	−	+	−
Li et al.^19^	+	+		−	+	+	−
Liu et al.^20^	+	+		−	+	+	−
Meng et al.^21^	+	+		+	+	+	+
Meng et al.^22^	+	+		+	+	+	+
Ouyang et al.^23^	+	+		+	+	+	+
Pan et al.^24^	+	?		−	+	+	−
Wang and Ge^25^	+	?		−	+	+	−
Xie et al.^26^	+	+		+	+	+	+
Yuan et al.^27^	+	?		−	+	+	−
Zhang et al.^28^	+	+		−	+	+	−
Zunlong et al.^29^	+	?		−	+	+	−
**DAA vs. PA**							
Barrett et al.^30^	+	−		?	?	+	−
Bon et al.^31^	+	+		+	+	+	+
Cheng et al.^32^	+	+		+	+	+	+
Moerenhout et al.^33^	+	+		+	+	+	+
Rykov et al.^34^	+	+		−	+	+	−
Taunton et al.^35^	+	+		?	+	+	?
Zhao et al.^36^	+	+		+	+	+	+

DAA: direct anterior approach; PA: posterior and posterolateral approaches; ( +): low risk of bias; (?): some concerns; (**−**): high risk of bias.

**Table 3:** Level of evidence assessment according to GRADE recommendations.

No. of studies	Design	Risk of bias	Inconsistency	Indirectness	Imprecision	Other considerations	Quality of evidence
**SuperPATH vs. PA**							
*HHS 3 months postoperatively*							
9	RCT	Serious	Serious	No serious indirectness	Serious	All studies were from China	Very low
*HHS 6 months postoperatively*							
8	RCT	Serious	No serious inconcistency	No serious indirectness	Serious	All studies were from China	Very low
*HHS 12 months postoperatively*							
6	RCT	Moderate	No serious inconcistency	No serious indirectness	Serious	All studies were from China	Low
*Acetabular cup anteversion angle*							
4	RCT	Moderate	Serious	No serious indirectness	Serious	All studies were from China	Very low
*Acetabular cup inclination angle*							
5	RCT	Moderate	No serious inconcistency	No serious indirectness	No serious imprecision	All studies were from China	Moderate
*Intraoperative blood loss*							
10	RCT	Serious	No serious inconcistency	No serious indirectness	Serious	All studies were from China	Very low
*Operation time*							
10	RCT	Serious	Serious	No serious indirectness	Serious	All studies were from China	Very low
*Incision length*							
11	RCT	Serious	No serious inconcistency	No serious indirectness	No serious imprecision	All studies were from China	Low
**DAA vs. PA**							
*HHS 3 months postoperatively*							
4	RCT	Moderate	Serious	No serious indirectness	Serious	–	Very low
*HHS 6 months postoperatively*							
3	RCT	Moderate	No serious inconcistency	No serious indirectness	Serious	–	Low
*HHS 12 months postoperatively*							
3	RCT	Moderate	No serious inconcistency	No serious indirectness	No serious imprecision	–	Moderate
*Acetabular cup anteversion angle*							
5	RCT	Serious	Serious	No serious indirectness	Serious	–	Very low
*Acetabular cup inclination angle*							
6	RCT	Moderate	No serious inconcistency	No serious indirectness	Serious	–	Low
*Intraoperative blood loss*							
3	RCT	Serious	No serious inconcistency	No serious indirectness	No serious imprecision	–	Low
*Operation time*							
5	RCT	Serious	Serious	No serious indirectness	No serious imprecision	–	Very low
*Incision length*							
2	RCT	Serious	No serious inconcistency	No serious indirectness	Serious	–	Very low

RCT: randomized controlled trial; HHS: Harris Hip Score; VAS: Visual Analog Scale.

### Clinical and statistical heterogeneity

The clinical characteristics for gender, age, and BMI (Table 1) showed no relevant differences between the patients in the experimental (either SuperPATH or DAA) and control group (PA). The statistical heterogeneity of all measured outcomes is shown in Figs. 2, 3, 4, 5, 6, 7, 8, 9.

**Figure 2:**
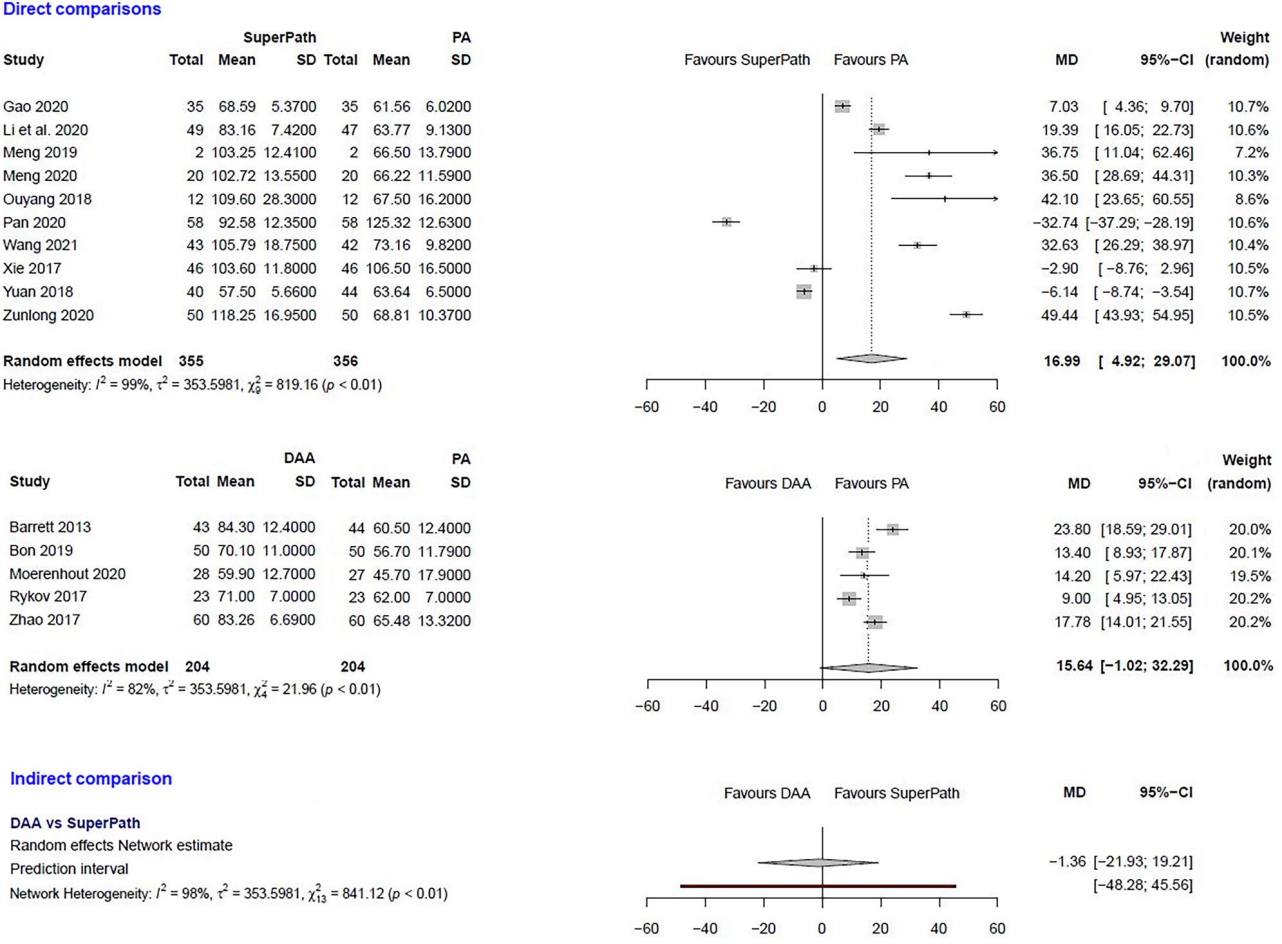
Comparison of the operation time in min. DAA: direct anterior approach; PA: posterior and posterolateral approaches; SD: standard deviation; MD: mean difference; CI: confidence interval.

**Figure 3:**
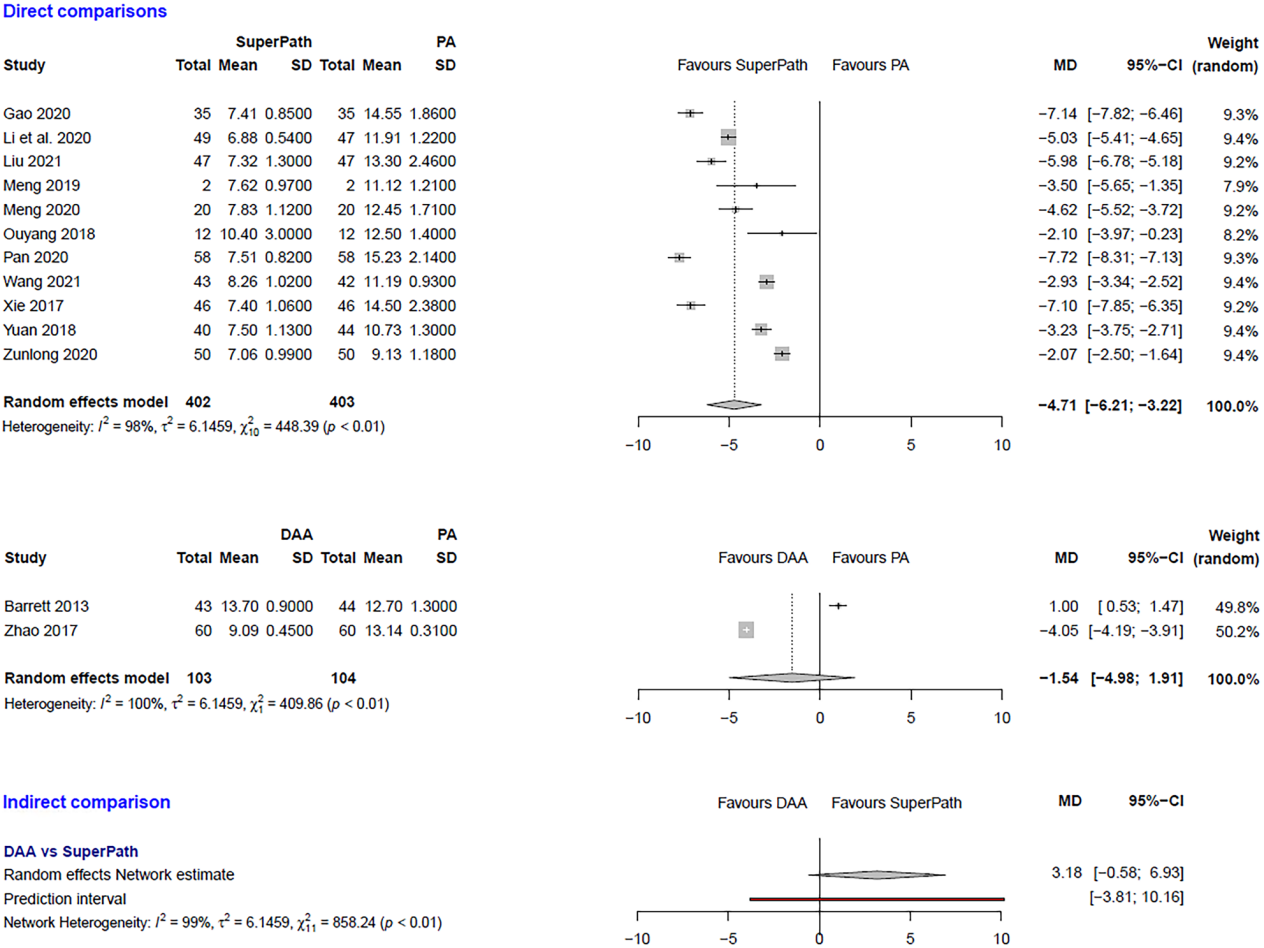
Comparison of the incision length in cm. DAA: direct anterior approach; PA: posterior and posterolateral approaches; SD: standard deviation; MD: mean difference; CI: confidence interval.

**Figure 4:**
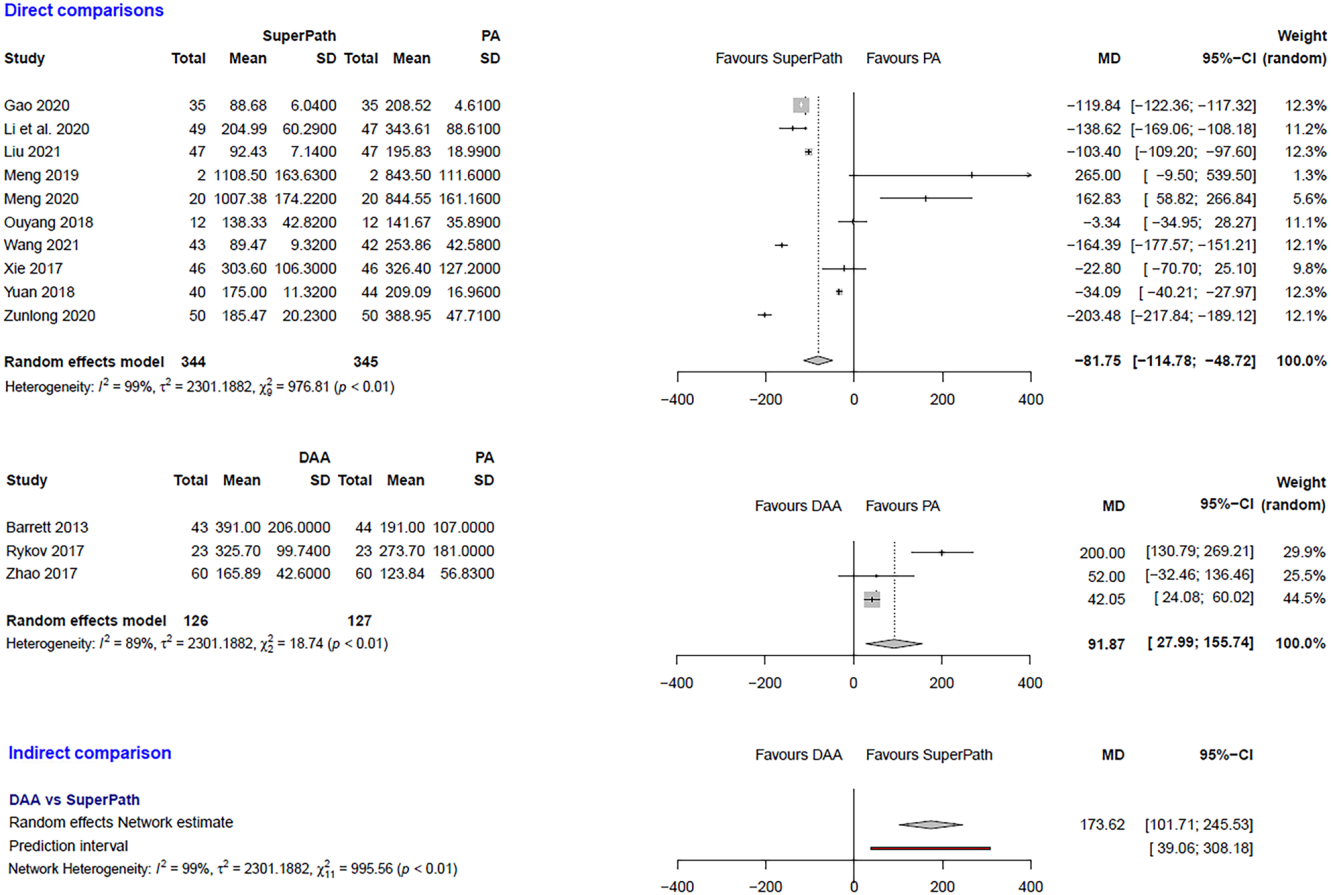
Comparison of the intraoperative blood loss in ml. DAA: direct anterior approach; PA: posterior and posterolateral approaches; SD: standard deviation; MD: mean difference; CI: confidence interval.

**Figure 5:**
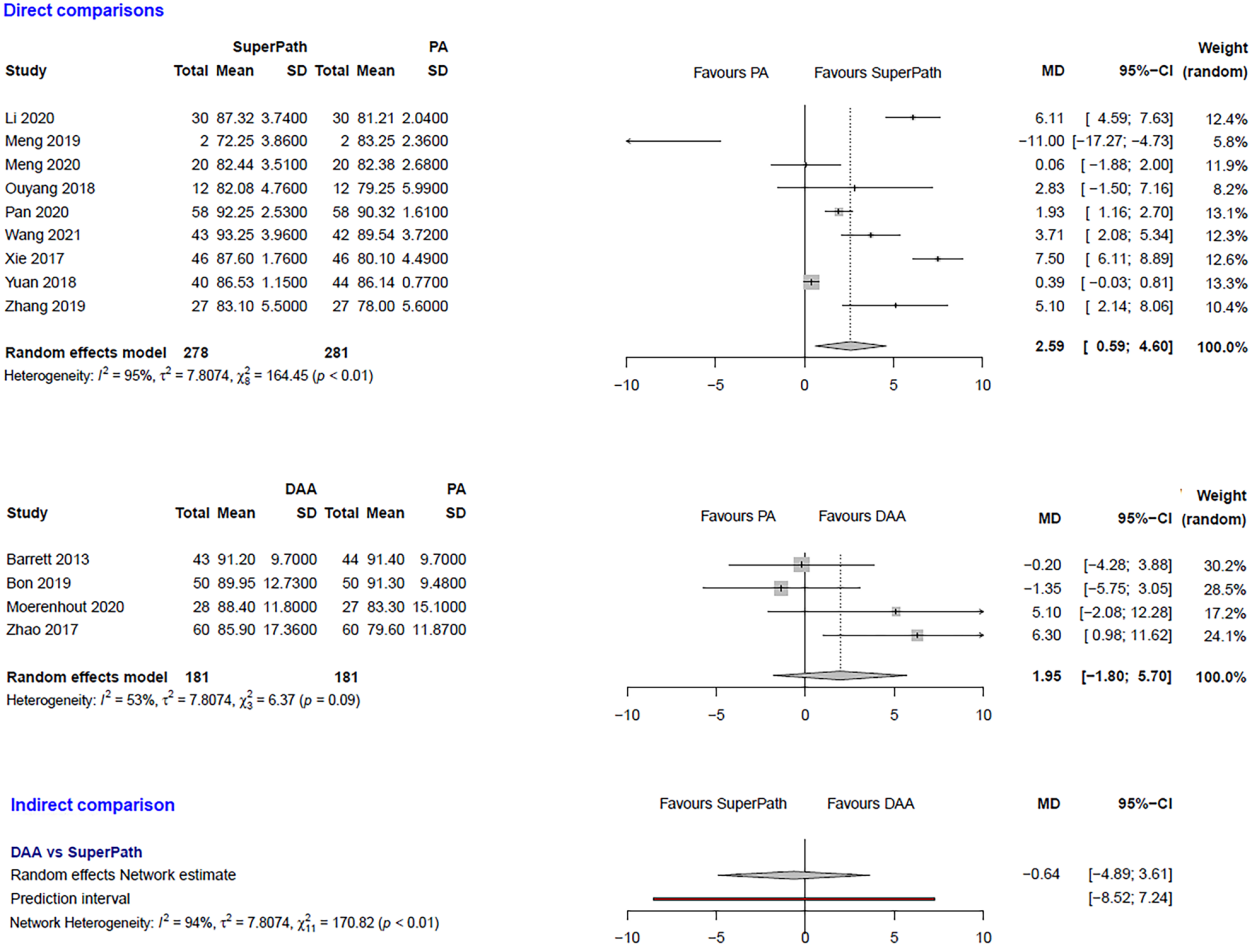
Comparison of the HHS 3 months postoperatively. DAA: direct anterior approach; PA: posterior and posterolateral approaches; SD: standard deviation; MD: mean difference; CI: confidence interval.

**Figure 6:**
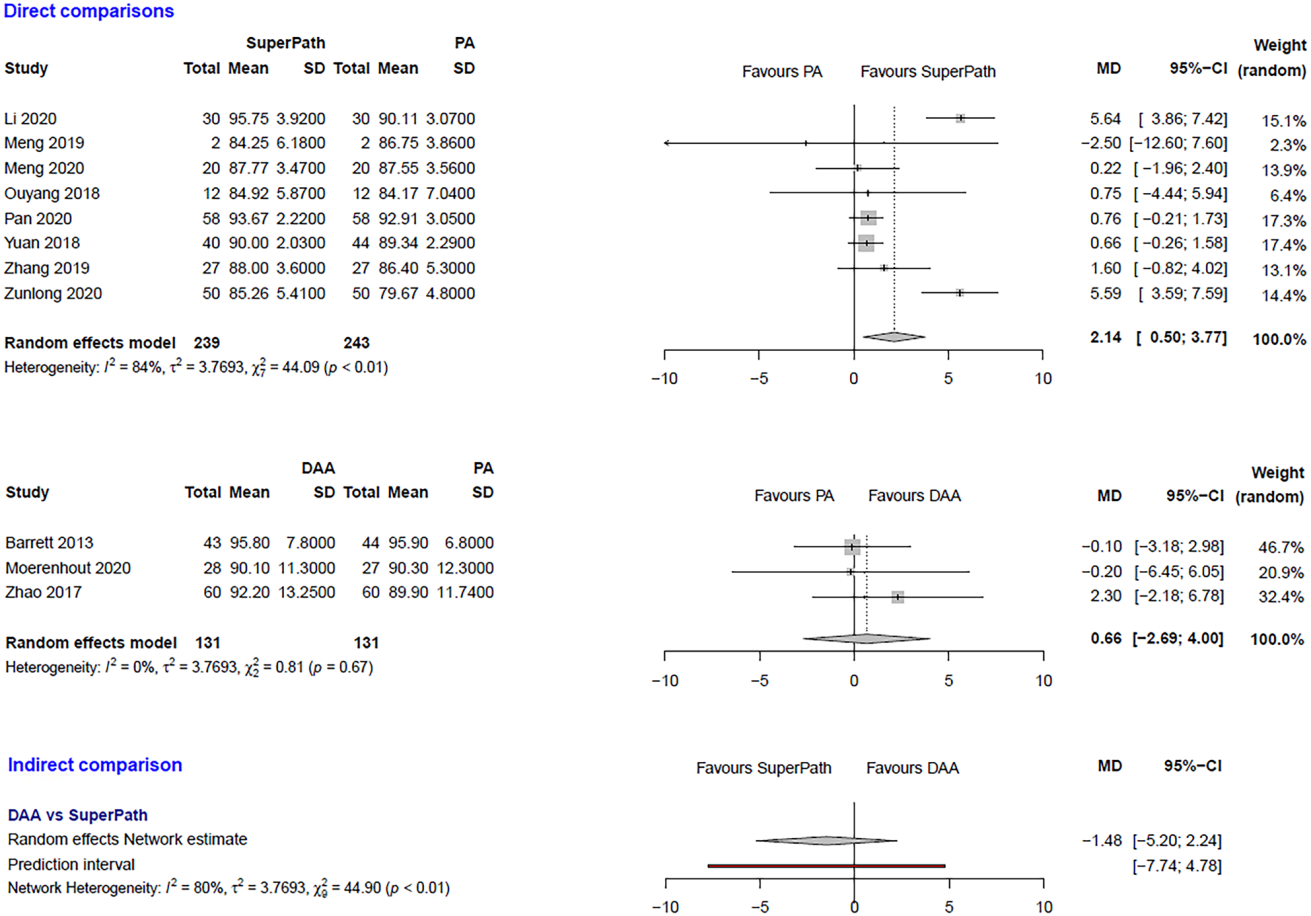
Comparison of the HHS 6 months postoperatively. DAA: direct anterior approach; PA: posterior and posterolateral approaches; SD: standard deviation; MD: mean difference; CI: confidence interval.

**Figure 7:**
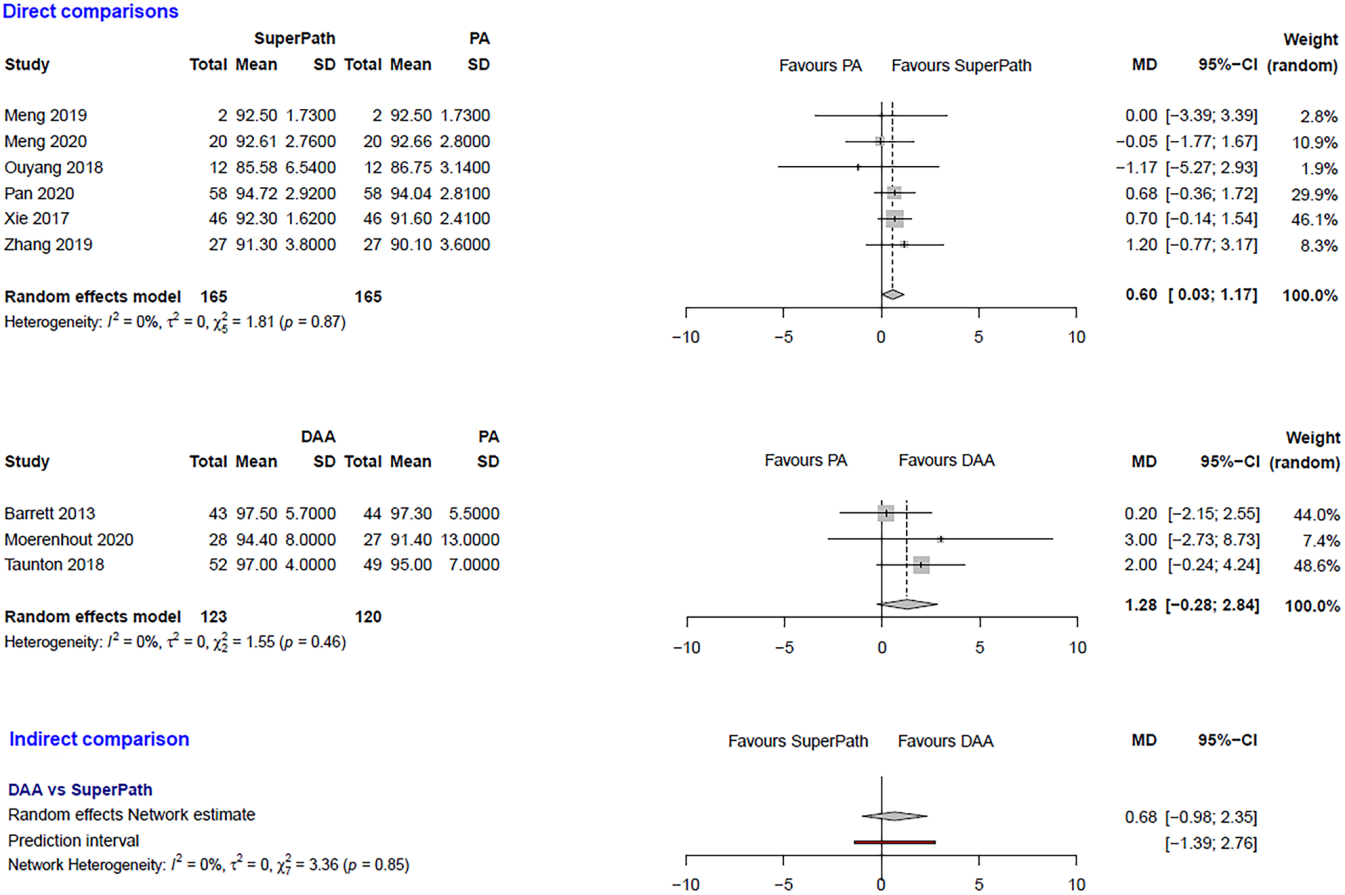
Comparison of the HHS 12 months postoperatively. DAA: direct anterior approach; PA: posterior and posterolateral approaches; SD: standard deviation; MD: mean difference; CI: confidence interval.

**Figure 8:**
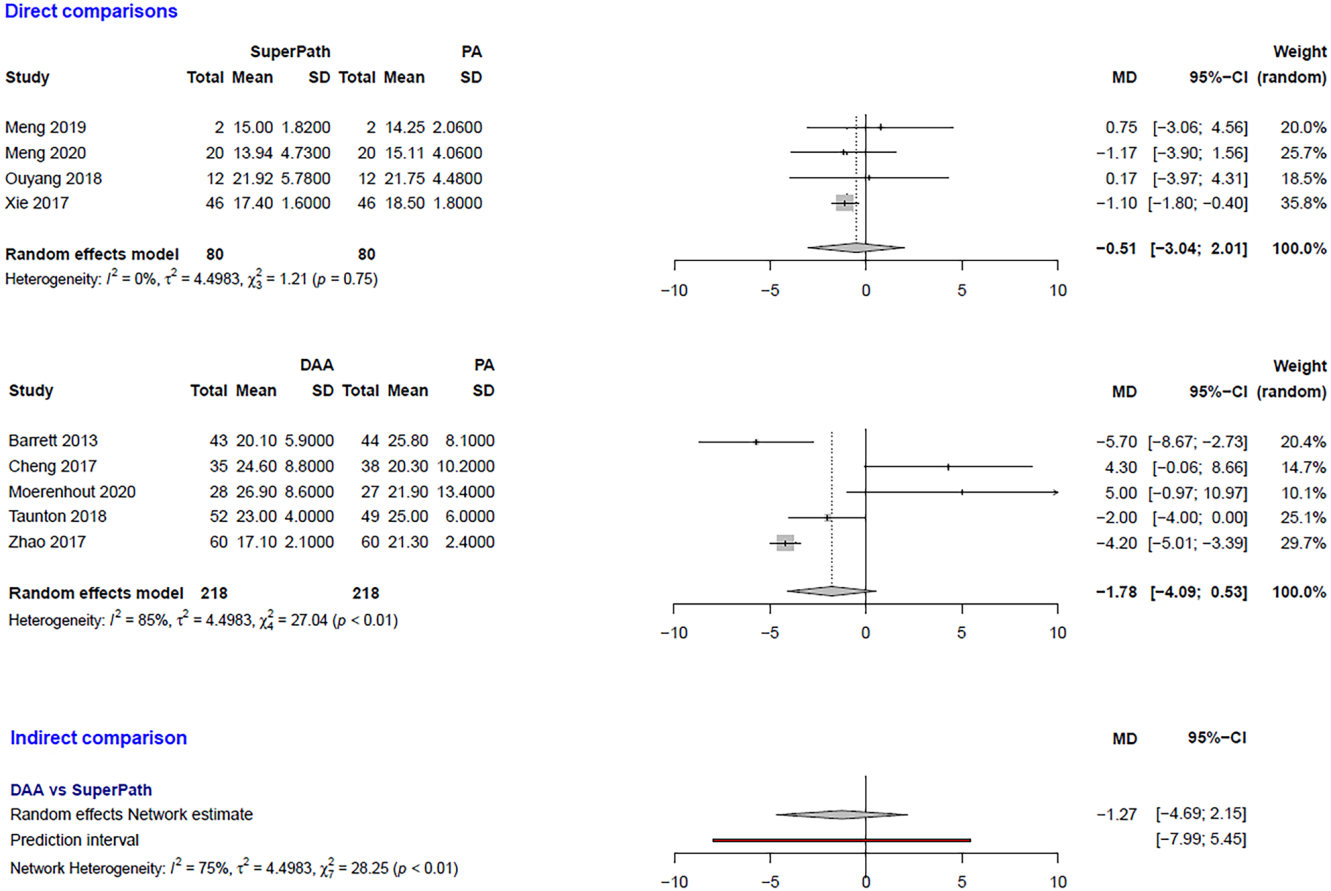
Comparison of the acetabular cup anteversion angle in degrees. DAA: direct anterior approach; PA: posterior and posterolateral approaches; SD: standard deviation; MD: mean difference; CI: confidence interval.

**Figure 9:**
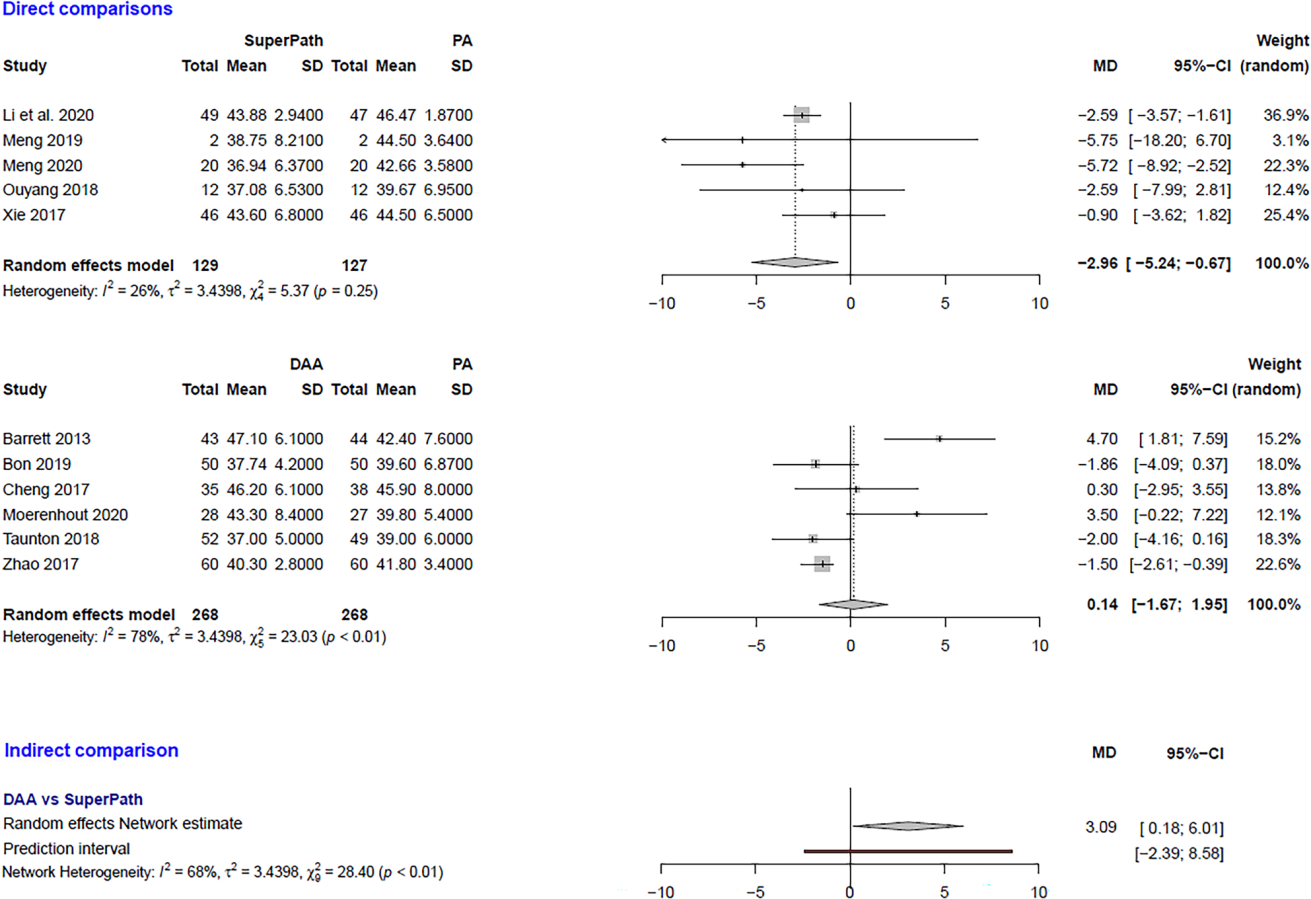
Comparison of the acetabular cup inclination angle in degrees. DAA: direct anterior approach; PA: posterior and posterolateral approaches; SD: standard deviation; MD: mean difference; CI: confidence interval.

### Outcomes

**1. Surgical outcomes**

***Operation time: SuperPATH vs. PA***

In a direct comparison between SuperPATH and PA, data on 711 patients were pooled from 10 RCTs (I^2^ = 99%, p < 0.01, Fig. 2). The operation time of SuperPATH was 17 min. longer than the operation time of PA (MD = 16.99, 95% CI 4.92 to 29.07).

***Operation time: DAA vs. PA***

In a direct comparison between DAA and PA, data on 408 patients were pooled from 5 RCTs (I^2^ = 82%, p < 0.01, Fig. 2). There was no difference in operation time (MD = 15.64, 95% CI  −1.02 to 32.29).

***Operation time: DAA vs. SuperPATH***

In an indirect comparison between DAA and SuperPATH, data on 559 patients were pooled from 15 RCTs (I^2^ = 98%, p < 0.01, Fig. 2). There was no difference in operation time (MD = −1.36, 95% CI  −21.93 to 19.21).

***Incision length: SuperPATH vs. PA***

In a direct comparison between SuperPATH and PA, data on 805 patients were pooled from 11 RCTs (I^2^ = 98%, p < 0.01, Fig. 3). The incision length of SuperPATH was 4.7 cm shorter than the incision length of PA (MD = −4.71, 95% CI −6.21 to −3.22).

***Incision length: DAA vs. PA***

In a direct comparison between DAA and PA, data on 207 patients were pooled from 2 RCTs (I^2^ = 100%, p < 0.01, Fig. 3). There was no difference in incision length (MD = −1.54, 95% CI −4.98 to 1.91).

***Incision length: DAA vs. SuperPATH***

In an indirect comparison between DAA and SuperPATH, data on 505 patients were pooled from 13 RCTs (I^2^ = 99%, p < 0.01, Fig. 3). There was no difference in incision length (MD = 3.18, 95% CI  −0.58 to 6.93).

***Intraoperative blood loss: SuperPATH vs. PA***

In a direct comparison between SuperPATH and PA, data on 689 patients were pooled from 10 RCTs (I^2^ = 99%, p < 0.01, Fig. 4). The intraoperative blood loss of SuperPATH was 81.8 ml less than the intraoperative blood loss of PA (MD = −81.75, 95% CI  −114.78 to −48.72).

***Intraoperative blood loss: DAA vs. PA***

In a direct comparison between DAA and PA, data on 253 patients were pooled from 3 RCTs (I^2^ = 89%, p < 0.01, Fig. 4). The intraoperative blood loss of DAA was 91.9 ml higher than the intraoperative blood loss of PA (MD = 91.87, 95% CI 27.99 to 155.74).

***Intraoperative blood loss: DAA vs. SuperPATH***

In an indirect comparison between DAA and SuperPATH, data on 470 patients were pooled from 13 RCTs (I^2^ = 99%, p < 0.01, Fig. 4). The intraoperative blood loss of DAA was 173.6 ml higher than the intraoperative blood loss of SuperPATH (MD = 173.62, 95% CI 101.71 to 245.53).

***2. Functional outcome: Harris Hip Score***

***HHS 3 months postoperatively: SuperPATH vs. PA***

In a direct comparison between SuperPATH and PA, data on 559 patients were pooled from 9 RCTs (I^2^ = 95%, p < 0.01, Fig. 5). The HHS 3 months postoperatively of SuperPATH was 2.6 points higher than the HHS 3 months postoperatively of PA (MD = 2.59, 95% CI 0.59 to 4.6).

***HHS 3 months postoperatively: DAA vs. PA***

In a direct comparison between DAA and PA, data on 362 patients were pooled from 4 RCTs (I^2^ = 53%, p = 0.09, Fig. 5). There was no difference in HHS 3 months postoperatively (MD = 1.95, 95% CI −1.8 to 5.7).

***HHS 3 months postoperatively: DAA vs. SuperPATH***

In an indirect comparison between DAA and SuperPATH, data on 459 patients were pooled from 13 RCTs (I^2^ = 94%, p < 0.01, Fig. 5). There was no difference in HHS 3 months postoperatively of DAA (MD = −0.64, 95% CI  −4.89 to 3.61).

***HHS 6 months postoperatively: SuperPATH vs. PA***

In a direct comparison between SuperPATH and PA, data on 482 patients were pooled from 8 RCTs (I^2^ = 84%, p < 0.01, Fig. 6). The HHS 6 months postoperatively of SuperPATH was 2.1 points higher than the HHS 6 months postoperatively of PA (MD = 2.14, 95% CI 0.5 to 3.77).

***HHS 6 months postoperatively: DAA vs. PA***

In a direct comparison between DAA and PA, data on 262 patients were pooled from 3 RCTs (I^2^ = 0%, p = 0.67, Fig. 6). There was no difference in HHS 6 months postoperatively (MD = 0.66, 95% CI −2.69 to 4.0).

***HHS 6 months postoperatively: DAA vs. SuperPATH***

In an indirect comparison between DAA and SuperPATH, data on 370 patients were pooled from 11 RCTs (I^2^ = 80%, p < 0.01, Fig. 6). There was no difference in HHS 6 months postoperatively (MD = −1.48, 95% CI  −5.2 to 2.24).

***HHS 12 months postoperatively: SuperPATH vs. PA***

In a direct comparison between SuperPATH and PA, data on 330 patients were pooled from 6 RCTs (I^2^ = 0%, p = 0.87, Fig. 7). The HHS 12 months postoperatively of SuperPATH was 0.6 points higher than the HHS 12 months postoperatively of PA (MD = 0.6, 95% CI 0.03 to 1.17).

***HHS 12 months postoperatively: DAA vs. PA***

In a direct comparison between DAA and PA, data on 243 patients were pooled from 3 RCTs (I^2^ = 0%, p = 0.46, Fig. 7). There was no difference in HHS 12 months postoperatively of DAA (MD = 1.28, 95% CI −0.28 to 2.84).

***HHS 12 months postoperatively: DAA vs. SuperPATH***

In an indirect comparison between DAA and SuperPATH, data on 288 patients were pooled from 9 RCTs (I^2^ = 0%, p = 0.85, Fig. 7). There was no difference in HHS 12 months postoperatively (MD = 0.68, 95% CI −0.98 to 2.35).

***3. Radiological outcome***

***Acetabular cup anteversion angle: SuperPATH vs. PA***

In a direct comparison between SuperPATH and PA, data on 160 patients were pooled from 4 RCTs (I^2^ = 0%, p = 0.75, Fig. 8). There was no difference in acetabular cup anteversion angle (MD = −0.51, 95% CI −3.04 to 2.01).

***Acetabular cup anteversion angle: DAA vs. PA***

In a direct comparison between DAA and PA, data on 436 patients were pooled from 5 RCTs (I^2^ = 85%, p < 0.01, Fig. 8). There was no difference in acetabular cup anteversion angle (MD = −1.78, 95% CI −4.09 to 0.53).

***Acetabular cup anteversion angle: DAA vs. SuperPATH***

In an indirect comparison between DAA and SuperPATH, data on 298 patients were pooled from 9 RCTs (I^2^ = 75%, p < 0.01, Fig. 8). There was no difference in acetabular cup anteversion angle (MD = −1.27, 95% CI −4.69 to 2.15).

***Acetabular cup inclination angle: SuperPATH vs. PA***

In a direct comparison between SuperPATH and PA, data on 256 patients were pooled from 5 RCTs (I^2^ = 26%, p = 0.25, Fig. 9). The acetabular cup inclination angle of SuperPATH was 3.0° lower than the acetabular cup inclination angle of PA (MD = −2.96, 95% CI −5.24 to −0.67).

***Acetabular cup inclination angle: DAA vs. PA***

In a direct comparison between DAA and PA, data on 536 patients were pooled from 6 RCTs (I^2^ = 78%, p < 0.01, Fig. 9). There was no difference in acetabular cup inclination angle (MD = 0.14, 95% CI −1.67 to 1.95).

***Acetabular cup inclination angle: DAA vs. SuperPATH***

In an indirect comparison between DAA and SuperPATH, data on 397 patients were pooled from 11 RCTs (I^2^ = 68%, p < 0.01, Fig. 9). The acetabular cup inclination angle of SuperPATH was 3.1° lower than the acetabular cup inclination angle of DAA (MD = 3.09, 95% CI 0.18 to 6.01).

## Discussion

### Main and new findings

Our NMA included 20 RCTs with 1501 patients. Of these, 7 RCTs involving 592 patients compared DAA with PA, and 13 RCTs involving 919 patients compared SuperPATH with PA. Our NMA indicated that the results of THA through SuperPATH were statistically superior to THA through PA regarding the investigated outcomes. SuperPATH showed statistically better results on incision length, intraoperative blood loss, and HHS than PA. SuperPATH showed statistically worse results in operation time than PA. DAA showed statistically worse results in intraoperative blood loss than PA. The other outcomes in THA through DAA and PA were indifferent. SuperPATH showed statistically better results in intraoperative blood loss than DAA. The other outcomes in THA through SuperPATH and DAA were indifferent. All approaches showed sufficient results in acetabular cup positioning.

The value of this NMA comes from the inclusion of RCTs and the employment of high-quality statistical methods. We performed the NMA with both a fixed and a random effects model. Our NMA is an attempt to overcome the limitations of our previous NMAs^2,3^ by systematically and quantitatively reviewing literature comparing SuperPATH, DAA, and PA.

### SuperPATH vs. DAA vs. PA

The mean operation time in our NMA ranged from 57 to 118 min. for SuperPATH, from 60 to 84 min. for DAA, and from 46 to 125 min. for PA. SuperPATH had a 17 min. longer operation time than PA. There was no difference in operation time between DAA vs. PA and SuperPATH vs. DAA. A prolonged operation time was found in other meta-analyses comparing SuperPATH with conventional approaches^37–39^. A 2018 meta-analysis by Wang et al.^40^ with 9 RCTs and 754 THAs showed no difference in operation time between DAA and PA. Wills et al. found that an operation time > 90 min. in THA leads to increased rates of superficial infections^41^. Surace et al. determined in an analysis of 89,802 THA cases that an optimal operation time of around 80 min. leads to a lower risk of perioperative complications^42^. The operational technique through SuperPATH and DAA is somewhat more complicated than through the conventional approaches. Because of this, SuperPATH and DAA have an extended learning curve for operating surgeons^43,44^. SuperPATH may have the potential for a shorter operation time as it is a novel approach.

The mean incision length in our NMA ranged from 6.9 to 10.4 cm for SuperPATH, from 9.1 to 13.7 cm for DAA, and from 9.1 to 15.2 cm for PA. SuperPATH had a 4.7 cm shorter incision length than PA. There was no difference in incision length between DAA vs. PA and SuperPATH vs. DAA. Several recent meta-analyses found a shorter incision length for SuperPATH compared to conventional approaches^37–39,45^, other meta-analyses found a shorter incision length for DAA compared to conventional approaches^40,46^. Both SuperPATH and DAA should aim for incision lengths of < 10 cm, as this is a requirement for minimally invasive hip surgery. As can be seen in the corresponding Forest plot, SuperPATH is more likely to meet this requirement. Nevertheless, a 2013 meta-analysis by Xu et al. with 14 RCTs and 1174 patients did not come to a definite overall conclusion on whether there is a relevant difference between mini-incision or standard incision in THA outcome^47^. On the other hand, a 2013 meta-analysis by Moskal et al. with 30 studies and 3548 THAs concluded that shorter incisions had a better short-term outcome after THA, compared to standard incisions^48^.

The mean intraoperative blood loss in our NMA ranged from 89 to 1108 ml for SuperPATH, from 166 to 391 ml for DAA, and from 123.8 to 844.6 ml for PA. SuperPATH had 82 ml lower intraoperative blood loss than PA. DAA had 92 ml higher intraoperative blood loss than PA and 174 ml higher intraoperative blood loss than SuperPATH. A lower blood loss for SuperPATH compared to conventional approaches was already found in earlier meta-analyses^37,39^. However, in our NMA DAA showed a higher blood loss than PA. A possible explanation is bleeding of branches of the lateral circumflex femoral artery, the ligation of which is sometimes problematic. Besides the approaches to the hip joint, other known factors that influence blood loss in hip surgery are the use of tranexamic acid and intraoperative active warming^49–51^.

The mean HHS 3 months postoperatively in our NMA ranged from 72.3 to 93.3 points for SuperPATH, from 85.9 to 91.2 points for DAA, and 78.0 to 91.4 points for PA. The mean HHS 6 months postoperatively in our NMA ranged from 84.3 to 95.8 points for SuperPATH, from 90.1 to 95.8 points for DAA, and from 79.7 to 95.9 points for PA. The mean HHS 12 months postoperatively in our NMA ranged from 85.6 to 94.7 points for SuperPATH, from 94.4 to 97.5 points for DAA, and from 86.6 to 97.3 points for PA. With regards to the postoperative functional outcome (HHS 3, 6, and 12 months postoperatively) SuperPATH demonstrated statistically superior results to PA. The difference in HHS decreased over time after surgery. SuperPATH had a 2.6 point higher HHS 3 months postoperatively, a 2.1 points higher HHS 6 months postoperatively, and a 0.6 points higher HHS 12 months postoperatively. This means that the strength of SuperPATH lies in the early functional outcome. However, when interpreting the results it is important to emphasize that differences in functional outcomes are not clinically relevant. The highest HHS difference noted in our NMA was 2.6 points at 3 months postoperatively. Although in the literature the minimal clinically important difference (MCID) for HHS varies according to different types of hip surgery^52–55^, it has been reported as no less than 7.9 points on the 0–100 HHS scale. There was no difference in HHS 3, 6, and 12 months postoperatively between DAA vs. PA and SuperPATH vs. DAA. Several meta-analyses on SuperPATH vs. conventional approaches came to similar conclusions^37–39,45^. In contrast to our findings, several meta-analyses showed better early functional results for DAA compared to conventional approaches^40,46,56^. However, HHS is a very important outcome parameter as it gives a comprehensive impression of the function of the operated hip.

The mean acetabular cup anteversion angle in our NMA ranged from 13.9° to 21.9° for SuperPATH, from 17.1° to 26.9° for DAA, and from 14.3° to 25.8° for PA. Each in another RCT, DAA^33^ and PA^30^ showed a slightly too large angle with 26.9° and 25.8°, respectively. The mean acetabular cup inclination angle in our NMA ranged from 36.9° to 43.9° for SuperPATH, from 37.0° to 47.1° for DAA, and from 39.6° to 46.5° for PA. In general, all approaches stayed within the widely accepted values for acetabular cup positioning: anteversion angle from 10° to 25° and inclination angle from 40° to 50°^9^. None of the included studies evaluated the restoration of the center of rotation of the native acetabulum, which could be affected during minimally invasive approaches, due to potential upward deviation of the acetabular reamers by the soft tissue tension. Furthermore, the assessment of the anteversion angle, in particular, is questionable, as none of the included RCTs used computed tomography (Table 1). In addition, both acetabular inclination and anteversion change between standing and supine conventional radiographs. Nevertheless, there was no relevant difference between SuperPATH, DAA, and PA in acetabular cup positioning.

Intra- and postoperative fractures, especially trochanteric fractures, infections, and hip dislocations are important complications that seem to show different patterns in certain approaches. Surgical revision rates, leg length discrepancies, and postoperative pain are also parameters often taken into consideration in comparisons of THA. Nevertheless, postoperative complications could not be compared due to the lack of consistent data in the RCTs included.

### Limitations

In this NMA we addressed an important limitation of our previous NMAs^2,3^: we distinguished between each conventional approach and compared SuperPATH, DAA, and PA. The other restrictions remain unchanged: First, due to the lack of RCTs that directly compare SuperPATH and DAA, we provided an indirect comparison of both approaches, which offers weaker evidence. Second, the long-term outcomes of THA were not considered. Third, due to insufficient data, important outcome parameters such as hospitalization time, postoperative drainage volume, postoperative pain, and complications could not be considered. Fourth, this NMA did not consider the possible influence of the surgeon operating skills, the utilization of tranexamic acid and anticoagulants, bone cement, or the types of implants for hip replacement. Fifth, part of the RCTs did not give any information on what exact hip pathology was treated with THA. Sixth, since the SuperPATH approach is a 2-incision approach, it remains unclear whether the included RCTs reported the added incision length or the length of the larger incision, ignoring the smaller additional incision. Lastly, in some cases of the outcomes investigated, the heterogeneity of the included RCTs was high.

## Conclusion

Our overall findings suggested that the short-term outcomes of THA through SuperPATH were statistically superior to PA. SuperPATH showed statistically better results in incision length, intraoperative blood loss, and functional outcome than PA. DAA and PA as well as SuperPATH and DAA showed overall indifferent short-term outcomes.

## Data Availability

The data are available from the corresponding author upon reasonable request.

## Abbreviations

BMI: Body mass index
CNKI: China National Knowledge Infrastructure
CI: Confidence interval
DAA: Direct anterior approach
HHS: Harris Hip Score
MD: Mean difference
NMA: Network meta-analysis
PA: Posterior and posterolateral approach
RCT: Randomized controlled trial
SuperPATH: Supercapsular percutaneously assisted approach in total hip arthroplasty
THA: Total hip arthroplasty
